# Optimal hemodynamic response model for functional near-infrared spectroscopy

**DOI:** 10.3389/fnbeh.2015.00151

**Published:** 2015-06-16

**Authors:** Muhammad A. Kamran, Myung Yung Jeong, Malik M. N. Mannan

**Affiliations:** Department of Cogno-Mechatronics Engineering, Pusan National UniversityBusan, Korea

**Keywords:** hemodynamic response model, physiological noises, functional near-infrared spectroscopy, optimization algorithm, brain imaging

## Abstract

Functional near-infrared spectroscopy (fNIRS) is an emerging non-invasive brain imaging technique and measures brain activities by means of near-infrared light of 650–950 nm wavelengths. The cortical hemodynamic response (HR) differs in attributes at different brain regions and on repetition of trials, even if the experimental paradigm is kept exactly the same. Therefore, an HR model that can estimate such variations in the response is the objective of this research. The canonical hemodynamic response function (cHRF) is modeled by two Gamma functions with six unknown parameters (four of them to model the shape and other two to scale and baseline respectively). The HRF model is supposed to be a linear combination of HRF, baseline, and physiological noises (amplitudes and frequencies of physiological noises are supposed to be unknown). An objective function is developed as a square of the residuals with constraints on 12 free parameters. The formulated problem is solved by using an iterative optimization algorithm to estimate the unknown parameters in the model. Inter-subject variations in HRF and physiological noises have been estimated for better cortical functional maps. The accuracy of the algorithm has been verified using 10 real and 15 simulated data sets. Ten healthy subjects participated in the experiment and their HRF for finger-tapping tasks have been estimated and analyzed. The statistical significance of the estimated activity strength parameters has been verified by employing statistical analysis (i.e., *t*-value > *t*_critical_ and *p*-value < 0.05).

## Introduction

Functional near-infrared spectroscopy (fNIRS) is a non-invasive and an emerging neuro-imaging technique (Santosa et al., 2013, 2014). The brain functional information is decoded through the interpretation of the variation in the optical properties of near-infrared (NIR) light (Naseer et al., 2014). NIRS monitors regional cerebral blood flow (rCBF) variations through the absorption changes of the NIR light at wavelengths between 650–950 nm. The oxy-hemoglobin (HbO) and deoxy-hemoglobin (HbR) are two major chromospheres in the blood which absorb NIR light (Cope and Delpy, 1988). The concentration of HbO and HbR varies in the capillary blood during the rest and task sessions (Hu et al., 2013). Thus, brain functional information can be revealed by the estimation of HbO and HbR. fNIRS, with the ability to estimate both chromospheres, is a potential brain imaging modality (Kamran and Hong, 2014). Functional-magnetic resonance imaging (fMRI) (Cohen et al., 2014; Zhou et al., 2014) and electro-encephalography (EEG) are most frequently used cortical imaging modalities in past. In comparison, the huge size of fMRI and its paramagnetic constraints and low spatial resolution of EEG (Soekadar et al., 2014) enhance the potential position of fNIRS with good temporal resolution applicable to brain-computer interface (BCI) applications (Hu et al., 2010). Its spatial resolution is affected by the low penetration depth but lies in between fMRI and EEG (Boudriay et al., 2014). In addition to these attributes, its portability, safety and low cost features makes it on top position for rehabilitation and BCI applications (Khan et al., 2014).

The detection of neuronal activation in a particular cortical area refers to find out a specific waveform in the hemodynamic response (HR) (Ciftçi et al., 2008). In hemodynamic related neuro-imaging modality, likewise fNIRS, the existence of such waveform is indicated as a statistical comparison to a specific time series shape, known as canonical hemodynamic response function (cHRF) (Abdelnour and Huppert, 2009). The cHRF has a key role in the analysis of fNIRS time series as its shape varies between different brain regions, repetition of trials, and among subjects as well (Hong and Nugyen, 2014). The difference in the dynamic shape of HRF during event-related motor and visual paradigms revealed that the peak times of HbO, HbR, and total hemoglobin (HbT) for visual paradigm are approximately equal unlike for motor paradigm (Jasdzewski et al., 2003). Additionally, it is found that the wavelength dependent differential path length factor (DPF) and age can also affect the characteristics of HR (Duncan et al., 1996). A mismatch between these features could result as a decrease in the detection performance (Ciftçi et al., 2008).

The most commonly used model for cHRF is composed of two gamma functions to characterize the shape and undershoot, respectively. It has been frequently be implemented in the analysis of fMRI temporal data. The performance accuracy of detection is improved by modifying the basis set, incorporating temporal derivative (TD) and dispersion derivation (DD) along with blood oxygen level dependent (BOLD) in the design matrix (Friston et al., 1998). Thus, modeled HR is represented as a linear combination of three waveforms. The characteristics of BOLD response are similar to cHRF used in NIRS data analysis. But fNIRS signal has additional challenge of temporal correlation present in the optical signal caused by physiological noises (Hu et al., 2010). The feature values for basis set were imposed constraints (time-to-peak, number of positive and negative peaks, time to- and magnitude of undershoot) to improve the extraction of the specific wave-pattern that formulate the dynamic shape of cHRF (Ciftçi et al., 2008). Finally, a general linear model (GLM) framework is being utilized to tune the unknown parameters in the model using Bayesian approach (Ciftçi et al., 2008). A new public statistical toolbox (NIRS-SPM freely available at http://bispl.weebly.com/nirs-spm.html#/) for the analysis of NIRS data was introduced in 2009, incorporating GLM based estimation of the cortical activity (Ye et al., 2009). NIRS-SPM is an extension of statistical parameter mapping toolbox for fMRI, thus it uses the GLM approach with basis set (Friston et al., 1998) to map the neuronal activities on brain templates. A detailed comparison of modeling techniques for HRF in fMRI regarding assumptions in the models, the complexity in their design and interpretation shows that it is difficult to accurately recover true task-evoked changes (Lindquist et al., 2009). In their study, the gradient approach has been utilized for the estimation of the free parameters that define the shapes of different HRF models. The fNIRS time series is contaminated with physiological noises. Thus, addition of physiological signals in the design matrix could improve the detection of task-related HR and its application to BCI (Abdelnour and Huppert, 2009). The parameters of cHRF were assumed to be fixed in Abdelnour and Huppert (2009) and activity strength parameters have been estimated using Kalman filters. The conventional averaging techniques have been used most frequently in past but its major drawback is the number of trials necessary to derive the stable HRF (Scarpa et al., 2010). Scarpa et al. (2013) proposed the methodology of near/closed channels to remove the physiological noises with fix parameters to extract cHRF. Several studies in past have presented the idea of combining HR model and adaptive signal processing algorithms to recursively tune the model parameters (Kamran and Hong, 2013; Santosa et al., 2013; Hong and Nugyen, 2014). Thus, an optimal HR model is still a topic of interest for many researchers in fNIRS area.

In this paper, an optimal HR model has been proposed for the analysis of fNIRS time series. The measured HR is modeled as a linear combination of evoked-HR, the physiological noises (cardiac pulsation, respiratory beat and low frequency Mayer waves) and base-line correction. The evoked-HR is the convolution of cHRF and the experimental paradigm. The cHRF has been modeled as a linear combination of two gamma functions (Lindquist et al., 2009). Six parameters in the cHRF model have been supposed as free parameters (delay of response relative to onset, delay of undershoot, dispersion of response, dispersion of undershoot, baseline, and a scaling factor) (Friston et al., 1994). The selection of optimal parameters in cHRF is the crucial step due to the variability of HR in different brain regions, repetition of trials and among subjects as well. In addition, the variation in the frequency and amplitudes of the physiological noises is a common phenomenon in the optical signal (Abdelnour and Huppert, 2009). Thus, these parameters in physiological noises are also supposed as free. The optimal parameters whose subsequent results best fit to the measured HR are found through an iterative optimization process. Initial values for these free parameters have been used from existing literature (Friston et al., 1994; Abdelnour and Huppert, 2009). Finally, the brain-activation model is formulated as an optimization problem with 12 free parameters. The formulated pre-optimized model is passed to an iterative simplex method with initial parameter vector. The simplex algorithm (Spendley et al., 1962) and its modified version (Nelder and Mead, 1965) has frequently been used in past for many signal processing and engineering-design optimization applications (Luersen and Riche, 2004). Fifteen simulated data sets have been generated with known parameters to verify the correctness of the proposed algorithm. Simulated data sets have been generated through method described in Prince et al. (2003). A low error in the estimation of free parameters shows great potential of the proposed algorithm in this field. Ten healthy participants have been examined for motor related, typically box-car based rest-task-rest experiment. Finally, brain functional maps have been shown to localize the cortical activity.

## Materials and methods

### Data acquisition and pre-processing

**Figure 1:**
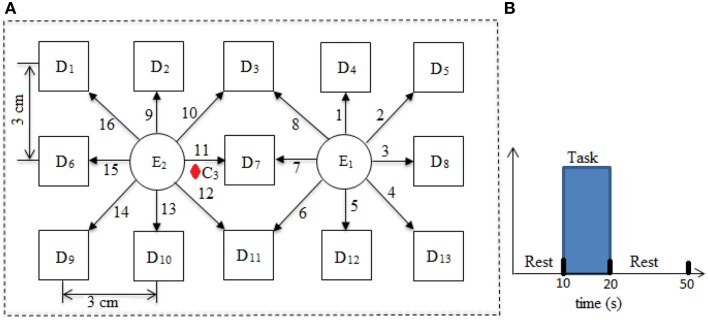
**Source/detector locations and distribution of channels (A) and experimental paradigm (B)**.

Three different types of optical neuro-imaging systems are available commercially, namely, continuous wave (CW), frequency domain (FD), and time-resolved spectroscopy (Hu et al., 2012; Schudlo et al., 2013). CW is the least expensive and most frequently used approach for BCI applications. It provides the relative change in the concentration of HbO and HbR. The CW-NIRS imaging system (DYNOT: Dynamic Near-infrared Optical Tomography; NIRx Medical Technologies, Brooklyn, NY) was used in this study with two wavelengths of NIR light (760 and 830 nm). It has 32 optodes which can be configured as emitters or detectors according to the experimental requirement with data acquisition frequency of 1.81 Hz. The optodes were placed on the left motor cortex at 16 different emitter–detector–pair locations. The source–detector separation was approximately 3 cm. The optode configuration has been shown in Figure 1A.

The optical density variations measured through NIRS imaging system is converted into relative concentration changes of HbO and HbR using modified-Beer Lambert law (MBLL). According to MBLL, the optical densities at two different wavelengths can be solved by simple algebra to estimate the relative concentration changes of HbO and HbR with assumption of constant scattering (Power et al., 2011; Kamran and Hong, 2013)

(1) $$\Delta HbO^{i}(k)=\frac{\left(\varepsilon_{HbR}^{\lambda_{1}}\frac{\Delta OD^{\lambda_{2}}(k)}{DPF^{\lambda_{2}}})-(\varepsilon_{HbR}^{\lambda 2}\frac{\Delta OD^{\lambda_{1}}(k)}{DPF^{\lambda_{1}}}\right)}{l^{i}(\varepsilon_{HbR}^{\lambda_{1}}\varepsilon_{HbO}^{\lambda_{2}}-\varepsilon_{HbR}^{\lambda_{2}}\varepsilon_{HbO}^{\lambda_{1}})},$$

(2) $$\Delta HbR^{i}(k)=\frac{\left(\varepsilon_{HbO}^{\lambda_{2}}\frac{\Delta OD^{\lambda_{1}}(k)}{DPF^{\lambda_{1}}})-(\varepsilon_{HbO}^{\lambda_{1}}\frac{\Delta OD^{\lambda_{2}}(k)}{DPF^{\lambda_{2}}}\right)}{l^{i}(\varepsilon_{HbR}^{\lambda_{1}}\varepsilon_{HbO}^{\lambda_{2}}-\varepsilon_{HbR}^{\lambda_{2}}\varepsilon_{HbO}^{\lambda_{1}})},$$

where Δ*HbO^i^* (*k*) and Δ*HbR*^*i*^ (*k*) are relative concentration changes of HbO and HbR, respectively, *k* is the discrete time, *i* represents the *i*th-channel of emitter-detector pair, λ_1_ and λ_2_ represent 760 and 830 nm wavelengths, ε^λ_1_^_*HbO*_, ε^λ_1_^_*HbR*_, ε^λ_2_^_*HbO*_, and ε^λ_2_^_*HbR*_ indicates the extinction coefficients (refers to the measure of absorption of light) of HbO and HbR at two different wavelengths, respectively, Δ*OD*^λ*j*^ (*k*) is the optical density variation at *k*th-sample time at particular wavelength (*j* = 1, 2), *l^i^* is the source-detector separation and *DPF*^λ^*j* is the DPF at particular wavelength (*j* = 1, 2). The extinction coefficients corresponding to 760 nm are 1.486 (for HbO) and 3.843 (for HbR) and those corresponding to 830 nm are 2.231 (for HbO) and 1.791 (for HbR) (Kamran and Hong, 2014).

### Experimental setup and paradigm

Ten right-handed healthy subjects (age: 28 ± 7 years) participated in this study. None of the subject has the neuronal-disorder history before the experimentation. The written consent of each participant was collected before experimentation. The experiment was in accordance with the latest version of the Declaration of Helsinki. The subjects were completely introduced about the experiment and instrument before the start of the experimentation. The subjects were advised to avoid the head motion as much as possible. The load of NIRS optode fibers were supported through the hanger available with the instrument. The experiment includes a typical box-car rest-task-rest session. The experiment includes an initial rest of 10 s followed by a task session of 10 s and 30 s of a rest at the end. The subjects were instructed to tap their right index finger during task. A monitor screen was placed in front of the subject at a distance of approximately 110 cm. It remained blank during rest sessions and showed “finger tapping” during task session. The experimental paradigm has been shown in Figure 1B.

### Simulated subjects data

The proposed algorithm is based upon an iterative optimization algorithm. Thus, it is necessary to verify the algorithm through simulated data sets with known values of free parameters. The simulated data is generated and supposed to be combination of HRF, three physiological signals, baseline term and random Gaussian noise. Fifteen different simulated data sets were generated using different values of free parameters. The data set has been generated with the methods described in existing literature (Prince et al., 2003; Abdelnour and Huppert, 2009)

(3) $$HRF(k)=h{\left(k\right)}^{*}u(k),$$

(4) $$\begin{array}{l} h(k)=\left[\frac{k^{\alpha_{1}-1}\beta_{1}^{\alpha_{1}}e^{-\beta_{1}k}}{\Gamma (\alpha_{1})}-\frac{k^{\alpha_{2}-1}\beta_{2}^{\alpha_{2}}e^{-\beta_{2}k}}{6\Gamma (\alpha_{2})}\right]\\ \; \end{array}$$

where *u* is the experimental paradigm, *h* is the cHRF, α_1_ is the delay of the response, α_2_ is the delay of the undershoot, β_1_ is the dispersion of the response, β_2_ is the dispersion of the undershoot and Γ represents the Gamma distribution. The physiological signals in simulated data have been generated through the linear combination of three sinusoids (Abdelnour and Huppert, 2009; Kamran and Hong, 2014). The specific values of free parameters used for all 15 data sets have been listed in Table 1.

**Table 1:** **The results of 15 simulated data-sets: the actual values of parameters (A) and estimated ones through the proposed algorithm (E)**.

**Sub**.	**A/E**	***a*_c_**	***f*_c_**	***a*_r_**	***f*_r_**	***a*_m_**	***f*_m_**	***α*_1_**	***α*_2_**	***ß*_1_**	***ß*_2_**	***a*_0_**	***a*_1_**
1	A	1	1	1	0.2	1	0.07	6	16	1	1	14	10
	E	1.44	0.81	0.90	0.24	0.72	0.01	6.01	15.97	1.00	0.99	13.99	10.07
2	A	1.1	0.9	1.2	0.3	1.1	0.09	7	15	0.8	0.7	12	8
	E	1.59	0.79	1.74	0.007	0.71	0.018	9.23	16.38	0.98	1.5	13.5	4.40
3	A	1.2	0.95	1.3	0.22	1.2	0.08	3	12	0.9	1.1	12	6
	E	0.85	1.33	1.75	0.25	1.49	0.009	2.99	11.63	0.90	1.06	12.00	6.14
4	A	0.9	1.1	1	0.24	0.9	0.07	8	7	1	1	9	7
	E	1.42	1.22	0.85	0.29	1.73	.009	8.40	15.56	1.02	0.03	7.50	6.99
5	A	0.8	1	1.3	0.25	0.8	0.06	5	11	0.7	1.1	12	5
	E	1.05	1.10	1.44	0.22	0.01	0.009	5.34	8.75	0.77	0.99	11.98	5.16
6	A	0.7	0.9	0.9	0.25	0.7	0.05	9	18	0.9	1.3	14	9
	E	1.32	1.05	1.12	0.06	0.11	0.01	8.96	18.24	0.89	1.31	14.00	9.034
7	A	0.5	0.95	0.7	0.29	0.6	0.06	3	8	0.6	0.2	12	9
	E	0.96	1.3	1.20	0.24	1.68	0.01	2.99	8.23	0.60	0.20	11.99	9.03
8	A	0.4	1.1	0.6	0.3	0.5	0.07	4	18	1	1	8	5
	E	0.37	0.74	1.53	0.27	0.60	0.02	4.00	18.18	1.00	1.00	7.99	5.06
9	A	0.2	0.9	1	0.2	0.3	0.08	6	16	0.6	1.2	7	4
	E	1.99	1.29	0.23	0.23	1.51	0.01	4.93	6.70	0.57	1.35	6.95	4.50
10	A	1.2	0.9	1.2	0.23	1	0.09	5.5	17	0.8	1.4	11	3
	E	0.93	0.91	0.77	0.23	0.78	0.01	5.50	16.98	0.80	1.39	10.99	3.00
11	A	1.2	0.8	1	0.24	1.1	0.02	7	12	1.1	1.2	15	5
	E	0.48	1.40	1.18	0.11	1.78	0.01	8.91	7.01	1.53	0.04	12.72	2.05
12	A	1.1	0.85	0.9	0.26	0.8	0.03	4	10	1	0.8	11	7
	E	1.59	0.60	1.89	0.27	0.28	0.01	3.99	9.92	1.00	0.78	11.00	6.94
13	A	0.6	1.2	0.8	0.28	0.9	0.07	8	12	1.3	1	9	4
	E	1.98	0.88	0.50	0.17	0.89	0.01	7.99	11.63	1.30	0.96	8.99	4.04
14	A	0.4	0.8	0.9	0.20	0.6	0.08	9	12	0.5	0.3	10	3
	E	1.81	1.49	0.68	0.02	1.55	0.09	9.99	15.39	0.58	1.02	11.44	2.71
15	A	0.9	0.7	1.1	0.25	1	0.06	7	18	0.7	1.5	12	5
	E	1.36	1.05	1.36	0.11	1.09	0.01	6.55	15.44	0.69	0.02	9.95	5.60

### Linear brain model and parameter optimization

GLM is a statistical linear model to decompose the output into predefined regressors. The existence of a particular regressor of interest depends upon the intensity of the activity strength parameter (Santosa et al., 2013). The positive value of the activity strength parameter with increasing *t*-value shows the significant existence of the particular regressor in the measured waveform (Hu et al., 2010). In this study, the measured HbO concentration change is supposed to be the linear combination of evoked-HRF, physiological noises and base-line correction

(5) $$\begin{array}{l} y_{HbO}^{i}(k)=a_{o}+a_{1}HRF(k)+a_{c}\sin(2\pi f_{c}k)+a_{r}\sin(2\pi f_{r}k)\\ \;+a_{m}\sin(2\pi f_{m}k)+\varepsilon^{i}(k), \end{array}$$

where *y*^i^_*HbO*_ is the measured HbO time series at *i*th-channel, *a*_*o*_ is the baseline, *a*_1_ is the activity strength parameter, *a*_*c*_, *a*_*r*_, *a*_*m*_, *f*_*c*_, *f*_*r*_, *f*_*m*_ are the amplitudes and the frequencies of the cardiac, respiratory and Mayer wave respectively and ε^*i*^ (*k*) is the zero mean Gaussian noise at *k*th-sample time. Let us define a cost function *J* as sum of squares of residuals

(6) $$\begin{array}{l} J=\sum_{k=1}^{N}\{y_{HbO}^{i}i(k)-(a_{o}+a_{1}HRF(k)+a_{c}\sin(2\pi f_{c}k)+\\ \;a_{r}\sin(2\pi f_{r}k)+a_{m}\sin(2\pi f_{m}k)){\}}^{2}. \end{array}$$

The above cost function can be formulated in an optimization environment with constraints

(7) $$\begin{array}{l} \min J(\alpha_{1},\alpha_{2},\beta_{1},\beta_{2},a_{o},a_{1},a_{c},a_{m},a_{r},f_{c},f_{r},f_{m})s.t\\ C_{1}:2\leq \alpha_{1}\leq 10,\;C_{7}:0\leq a_{c}\leq 2,\\ C_{2}:6\leq \alpha_{2}\leq 20,\;C_{8}:0\leq a_{r}\leq 2,\\ C_{3}:0.5\leq \beta_{1}\leq 2,\;C_{9}:0\leq a_{m}\leq 2,\\ C_{4}:0\leq \beta_{2}\leq 1.5,\;C_{10}:0.5\leq f_{c}\leq 1.5,\\ C_{5}:0\leq a_{o}\leq 20,\;C_{11}:0.2\leq f_{r}\leq 0.3,\\ C_{6}:0\leq a_{1}\leq 15.\;C_{12}:0.09\leq f_{m}\leq 0.1. \end{array}$$

The optimal values of free parameters (α^*^_1_,α^*^_2_,β^*^_1_,β^*^_2_,a^*^_o_,a^*^_1_,a^*^_c_,a^*^_m_,a^*^_r_,f^*^_c_,f^*^_r_,f^*^_m_) are estimated by improved version of simplex method [later named as Nelder–Mead simplex method (NMSM)]. The iteration of NMSM can be performed by three steps, namely, ordering, centroid and transformation. The simplex of size *a* is defined at initial point (Haftka et al., 1990)

(8) $$x_{j}=x_{o}+pe_{j}+\sum_{\begin{array}{l} k=1\\ k\neq j \end{array}}^{n}qe_{k};j=1,2,\ldots ,n,$$

(9) $$p=\frac{a}{n\sqrt{2}}(\sqrt{n+1}+n-1)\text{ \& }q=\frac{a}{n\sqrt{2}}(\sqrt{n+1}-1).$$

where *x*_*j*_ (*j* = 1, 2,.., *n*) represent the vertices, *x*_*o*_ is the initial guess, *n* = 12 in this study and represents the number of free parameters, *e*_*j*_ represents the unit vector in the direction of *j*th vertex. The next step is to order the function in increasing order at all vertices and it is easy to sort as

(10) $$J\text{(}x_{l}\text{) < }J\text{(}x_{s}\text{) < }J\text{(}x_{h}\text{).}$$

where *x*_*l*_, *x*_*h*_, and *x*_*s*_, are the vertices with minimum value, maximum value and second highest value of the cost function, respectively. The next step is to discard the highest value by defining the centroid

(11) $$\bar{x}=\frac{1}{n}\sum_{\begin{array}{l} i=0\\ i\neq h \end{array}}^{n}x_{i},$$

where *x* is the centroid. The replacement of upper bound vertex of the cost function is done by reflection, expansion, contraction, and shrinkage (Lagarias et al., 1998). The mathematical equations for all these steps are given below

(12) $$\text{Reflection}:x_{r}=\bar{x}+\delta_{1}(x_{h}-\bar{x}),$$

(13) $$\text{Expansion}:x_{e}=\bar{x}+\delta_{2}(x_{r}-\bar{x}),\;$$

(14) $$\text{Contraction}:x_{c}=\bar{x}+\delta_{3}(x_{h}-\bar{x}),\;$$

(15) $$\text{Shrinkage}:x_{e}=\bar{x}+\delta_{4}(x_{l}-x_{i});\;i=0,1,\ldots ,n,$$

where δ_1_, δ_2_, δ_3_, and δ_4_ are coefficients of reflection, expansion, contraction, and shrinkage, respectively. The typical values of these coefficients have been chosen as 1, 2, 0.5, and 0.5, respectively (Lagarias et al., 1998; Luersen and Riche, 2004). The schematic of the algorithm is shown in Figure 2. The updated value at any step of iteration shall be replaced with bounded value, if it crosses bound at any step. The detail about the algorithm can be found in Haftka et al. (1990), Lagarias et al. (1998), and Luersen and Riche (2004).

**Figure 2:**
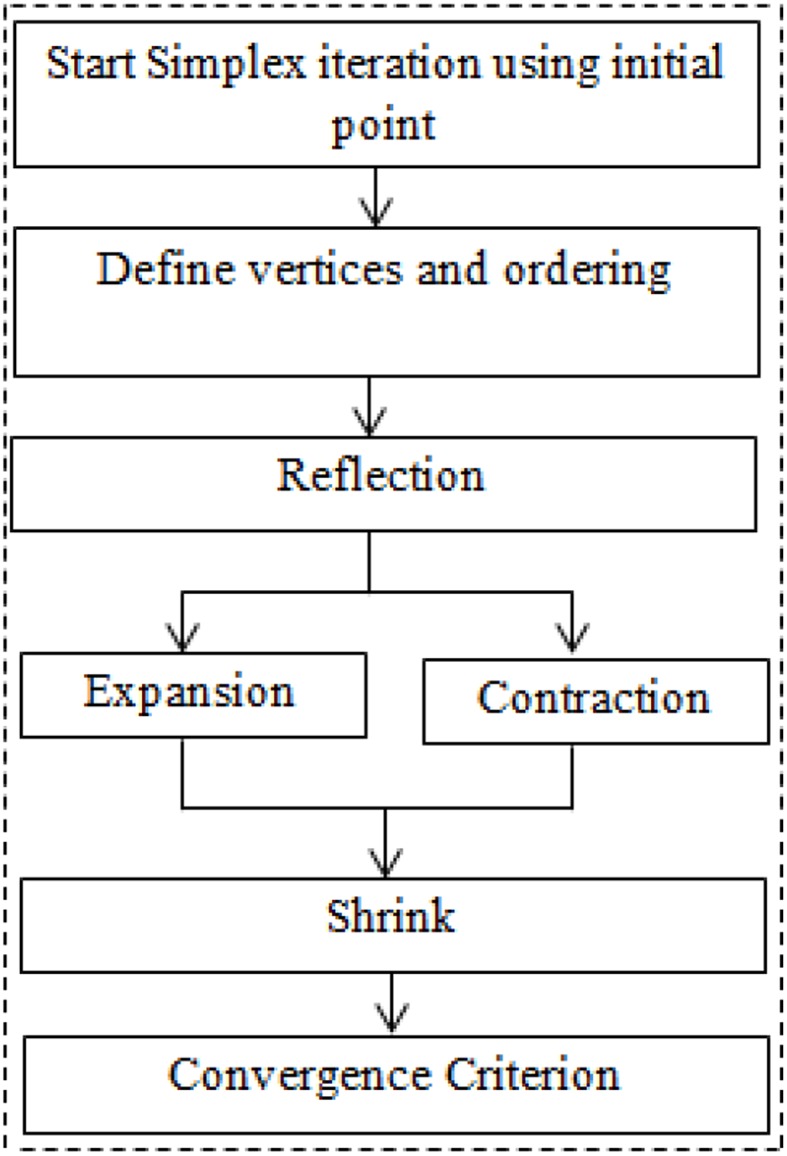
**Schematic of Nelder–Mead simplex method**.

### Functional brain maps and statistical significance

The estimation of the cortical activation and its localization is a challenging task in the analysis of fNIRS data series. Previous studies showed that localization of the cortical activation could be statistically estimated by fitting the estimated HRF to a pre-defined HRF (Hu et al., 2010; Kamran and Hong, 2014; Santosa et al., 2014). Let the optimal brain activation model is

(16) $$\begin{array}{l} y_{HbO}^{i}(k)=a_{0}^{*}+a_{1}^{*}HRF^{*}(k)+a_{c}^{*}\sin(2\pi f_{c}^{*}k)+a_{r}^{*}\sin(2\pi f_{r}^{*}k)\\ \;+a_{m}^{*}\sin(2\pi f_{m}^{*}k)+\varepsilon^{i}(k), \end{array}$$

The estimated optimal value of the activity strength parameter, a_1_, related to HRF indicates the activation of the particular brain region with proper statistics (Hu et al., 2010). The basic idea is to test whether the estimated value of the activity strength parameter is greater or less than a target value zero with statistically significance (*t*-value > *t*_critical_ and *p*-value < 0.05). Thus, it is equivalent of testing a null hypothesis *H_o_* with proper statistics i.e.,

(17) $$H_{o}:a_{1}^{*}=0$$

(18) $$t_{value}=\frac{a_{1}^{*}-0}{SE(a_{1}^{*})}.$$

where SE is the standard error of the estimated coefficient.

## Results

**Figure 3:**
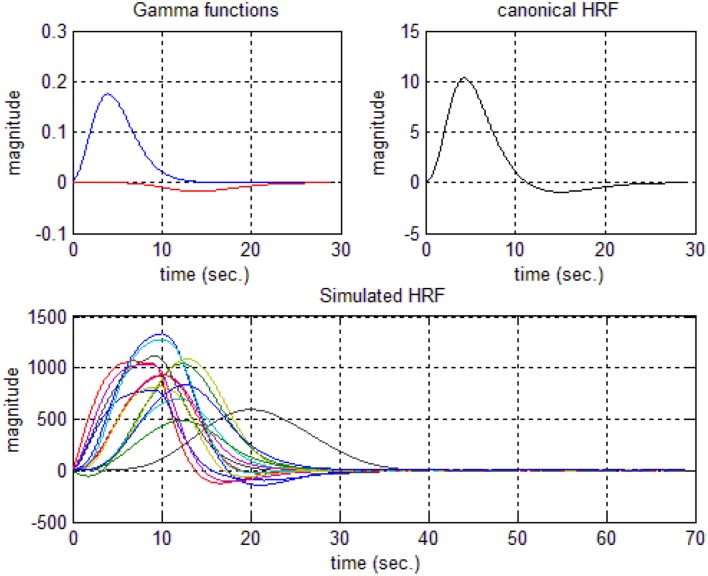
**Hemodynamic response function generations: two Gamma functions for generation of cHRF (top left), the standard cHRF (top right) and different simulated HRF (bottom)**.

**Figure 4:**
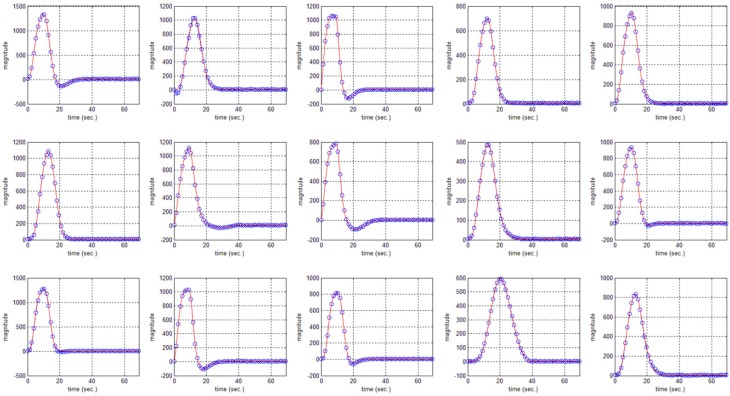
**HRF using actual values of free parameter (solid) and HRF using estimated values of free parameter (circular blue)**.

**Figure 5:**
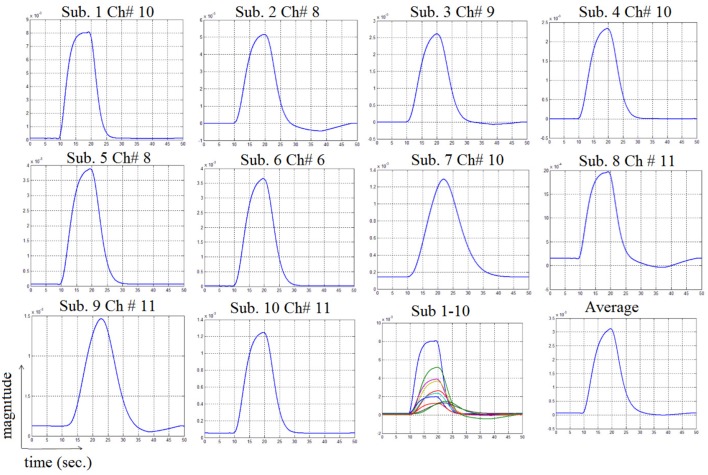
**Results of estimated HRF related to most active channel corresponding to all subjects**.

**Figure 6:**
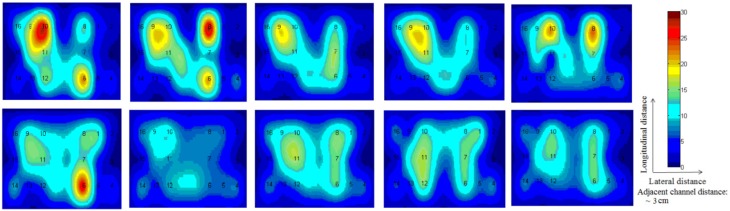
***t*-Maps of each subject and all channels**.

**Figure 7:**
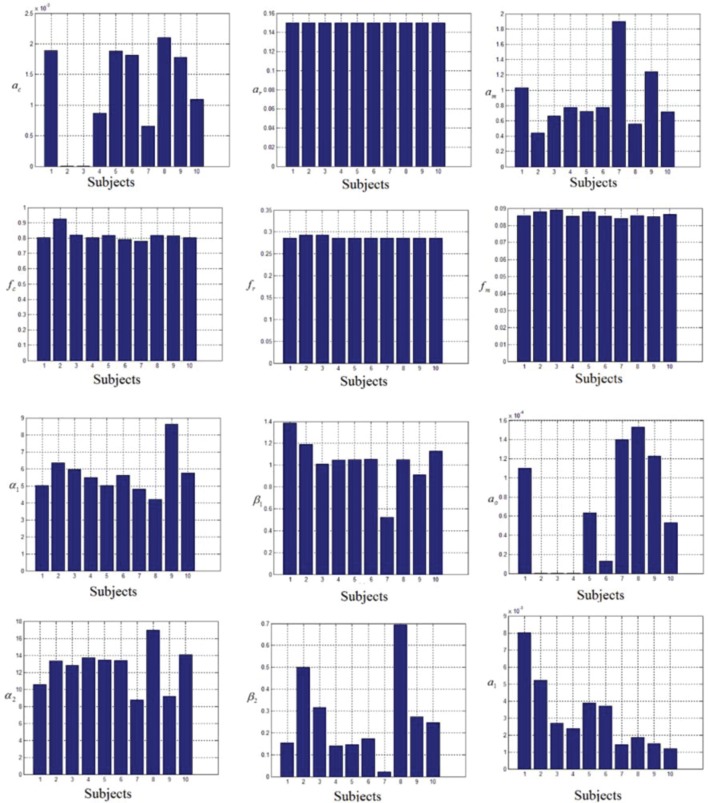
**Variations in the estimated parameters in real data sets**.

**Figure 8:**
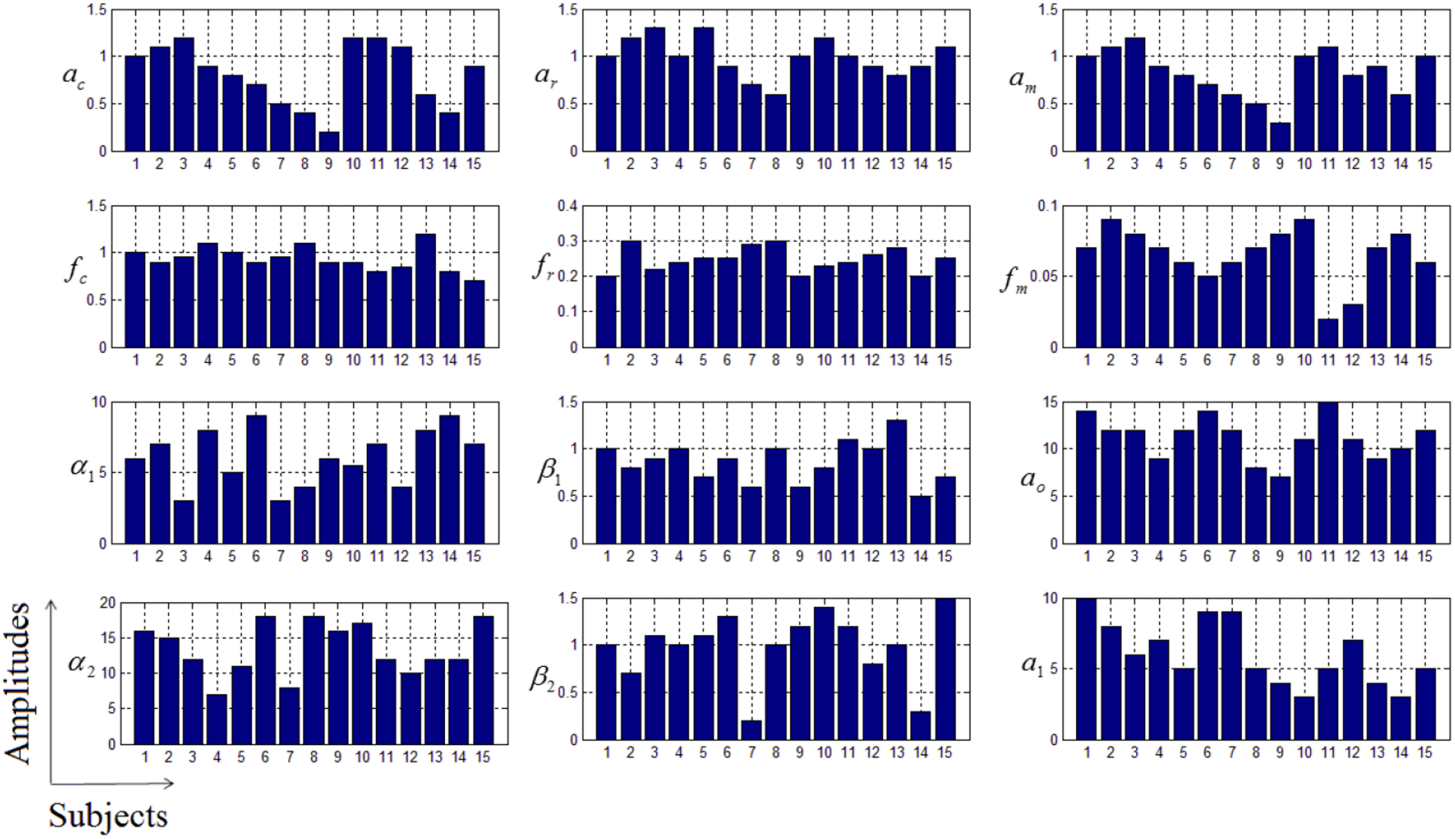
**Variations in the estimated parameters in simulated data sets**.

In this study an online recursive optimization algorithm is proposed for cortical activation detection to display discrete brain maps. The algorithm is verified through 15 synthetic data sets and implemented to real data sets of 10 healthy subjects. The cHRF (Figure 3, top right panel) is modeled as two Gamma functions (Figure 3, top left panel). All the simulated data sets have been displayed in Figure 3 (bottom panel). It is obvious to note that different width, height and undershoot have been considered for verifications. Table 1 summarized the values of free parameters and their estimate through proposed algorithm. The comparison of HRF with actual parameter values and estimated parameter values has been shown in Figure 4. The results of the estimated-evoked-HR of 10 subjects have been presented in Figure 5. The significance of results has been verified using *t*-test. The *t*-maps of the cortical activations have been presented in Figure 6. Figures 7, 8 display a comparison of the estimated parameters related to most active channel in each subject of real data set and simulated data set, respectively.

## Discussion

The non-invasive neuro-imaging techniques have a favorable position due to an increasing demand of BCI applications in the rehabilitation and medical diagnostics. There are several studies reported for the estimation of HRF in fMRI with numerical optimization techniques (Lindquist et al., 2009; Shah et al., 2014). But in the case of fNIRS, it constitutes an additional challenge of the physiological noise in the optical signal. Recently, several studies have been reported to analyze fNIRS time series using existing/new and/or modified versions of existing HRF models (Abdelnour and Huppert, 2009; Hu et al., 2010; Kamran and Hong, 2013, 2014; Santosa et al., 2013; Scarpa et al., 2013; Hong and Nugyen, 2014). The approaches vary in their implementation from simple estimation algorithms to more complex adaptive algorithms (Kamran and Hong, 2013) and blind signal processing (Santosa et al., 2013).

Hu et al. (2010) decomposed measured HRF into predefined regressors (evoked-HRF, base line correction and three others were included to design a set of high pass filter). Santosa et al. (2013) implemented the independent component analysis (ICA) framework to extract the statistically significance of a known wave pattern in the observed fNIRS data. Kamran and Hong (2013) explored the idea of adaptive signal processing to tune the variations in the measured HRF using parameter varying methodology. Later Kamran and Hong (2014) proposed to decompose HbO signal using ARMAX model for better cortical estimation as compared to existing ones. Abdelnour and Huppert (2009) proposed the adaptive framework to tune the HR with pre-built HRF in the model. Scarpa et al. (2013) emphases to remove the physiological noises by incorporating a near-detector (<0.7 cm from source) and to model the remaining signal as linear combination of pre-set evoked-HR and base line in Bayesian framework for adaptive tuning.

The DPF is a wavelength dependent factor and also varies with age of the subject causing variations in the hemodynamic signal (Duncan et al., 1996). Jasdzewski et al. (2003) reported that difference found in the characteristics of HRF in multiple brain regions. Their results suggest that the characteristics of HRF in different brain regions show variations. It is also observed in their study that some of the features like initial dip could be found in certain brain regions but not all. Hong and Nugyen (2014) developed a state-space model for different brain regions using adaptive signal processing framework. Their results revealed that there exist a significant difference between responses of different brain regions. It is a well-known fact that the hemodynamic signal has inter-subject variability as well as inter-trial variations. Hu et al. (2013) analyzed the reduction of trial-to-trial variations by analyzing correlation in the observed signal of different channels.

Thus existing literature suggest that the HR varies in its shape and characteristics not only in different brain areas, but it differs corresponding to the different mental task complexity, repetition, inter-subject and inter-trials as well. Some fNIRS-based BCI studies suggest that learning can improve the response, that is, less effort is required to repeat the same mental task. Thus, it is very important to model the HR in an adaptive framework together with a setup in which the parameters of HRF could be optimized as per real-time information in the measured response. Therefore, a recursive optimization algorithm have been presented in this study to model the variations in the HR. In contrast to existing fNIRS data analysis models, the proposed model has the capability to track time-varying characteristics (if exist) of HRF within same experiment as well. The estimation of pre-defined parameters of simulated data is shown in Table 1. It is obvious to observe that the proposed model estimated these parameters with a significant accuracy. After the validation of the algorithm using simulated data set, the methodology is applied to real data set of 10 subjects. The optimized values of HRF model parameters for 10 subjects have been listed in Table 2. The *t*-maps of each subject and all channels have been presented in Figure 6. It is evident from Figures 5, 6 that inter-subject difference exist in HRF parameters. Generally, inter-subject variability is due to the individual's differences in anatomical factors likewise skull and cerebrospinal fluid (CSF) structure, vessels distributions and the ratios of the arteries and veins. Thus, some of the subjects have more activation as compared to others.

**Table 2:** **The values of free parameter estimated through proposed algorithm in most active channel of each subject**.

**Sub**.	***a*_c_**	***f*_c_**	***a*_r_**	***f*_r_**	***a*_m_**	***f*_m_**	***α*_1_**	***α*_2_**	***β*_1_**	***β*_2_**	***a*_0_**	***a*_1_**
1	0.00189	0.80311	0.15	0.28625	1.02993	0.08562	5.00065	10.5287	1.38593	0.15405	0.00011	8.02E-5
2	1.39E-10	0.92423	0.15	0.29274	0.44333	0.08792	6.35815	13.3583	1.19083	0.49997	5.41E-12	5.23E-5
3	7.57E-10	0.82143	0.15	0.29286	0.66165	0.08904	5.95863	12.8180	1.00771	0.31555	2.78E-13	2.69E-05
4	0.000866	0.803147	0.15	0.286365	0.775247	0.085313	5.497915	13.69872	1.044254	0.140721	6.68E-12	2.38E-05
5	0.0018793	0.815845	0.15	0.28643	0.72472	0.087949	5.020148	13.44807	1.051638	0.146827	6.34E-05	3.87E-05
6	0.0018195	0.790466	0.15	0.286283	0.773555	0.085377	5.63	13.39223	1.054951	0.172463	1.29E-05	3.71E-05
7	0.0006532	0.778724	0.15	0.286524	1.900005	0.08407	4.811197	8.741103	0.521305	0.02237	0.00014	1.43E-05
8	0.0020988	0.816158	0.15	0.286284	0.560308	0.085565	4.190606	16.95479	1.050989	0.694504	0.000153	1.85E-05
9	0.0017808	0.815718	0.15	0.286434	1.245347	0.085161	8.609862	9.202103	0.910313	0.273311	0.000123	1.5E-05
10	0.001092	0.803117	0.15	0.286282	0.71349	0.086521	5.750496	14.08988	1.127646	0.245757	5.33E-05	1.21E-05

## Conclusion

An optimal HR model has been proposed that can extract the shape and scale of the HRF in addition to the amplitudes and the frequencies of the physiological sinusoids. Twelve parameters in the HR model have been supposed free with bounded constraints. The problem is formulated as an optimization problem and solved through an iterative optimization framework. The algorithm is first verified through different simulated data sets with known values of free parameters. A low error in estimation shows the accuracy of the proposed methodology. Furthermore, the algorithm is implemented to real data sets of 10 healthy participants. The parameters in HRF while repeating same trials are found to be different. Thus, it shall be beneficial for fNIRS data analysis, as the proposed model can track the characteristics of changes in HRF and physiological noises.

### Conflict of interest statement

The authors declare that the research was conducted in the absence of any commercial or financial relationships that could be construed as a potential conflict of interest.
